# CRISPR/Cas9-Based Modeling of *JAK2* V617F Mutation in K562 Cells Reveals Enhanced Proliferation and Sensitivity to Therapeutic Agents

**DOI:** 10.3390/ijms26104600

**Published:** 2025-05-11

**Authors:** Nungruthai Nilsri, Rujira Mekchaaum, Supaporn Kalasin, Jirapas Jongjitwimol, Krai Daowtak

**Affiliations:** 1 Department of Medical Technology, Faculty of Allied Health Sciences, Naresuan University, Phitsanulok 65000, Thailand; nungruthaini@nu.ac.th (N.N.); rujira.looknam@gmail.com (R.M.); supaporn4046@gmail.com (S.K.); jirapasj@nu.ac.th (J.J.); 2 Cellular and Molecular Immunology Research Unit, Faculty of Allied Health Sciences, Naresuan University, Phitsanulok 65000, Thailand

**Keywords:** *JAK2* V617F mutation, CRISPR/Cas9, interferon α2a, arsenic trioxide

## Abstract

The Janus kinase 2 (JAK2) protein fulfills an important role in hematopoiesis via the Janus kinase/signal transducer and activator of transcription (JAK/STAT) pathway, as it provides the genetic driver of *BCR::ABL1*-negative myeloproliferative neoplasms (MPNs), which are clinically manifested as polycythemia vera (PV), essential thrombocythemia (ET), and primary myelofibrosis (PMF). The most common cause of MPNs is the mutation of *JAK2* V617F in the *JAK2* gene, which results in increased cell proliferation. However, both the pathogenesis and treatment regimen of *BCR::ABL1*-negative MPNs remain poorly understood. The aim of the present study was to establish K562 cell lines with a point mutation in exon 14 (*JAK2*p.V617F) using CRISPR/Cas9 technology. The modified *JAK2* V617F cell lines were examined for the gene mutation using droplet digital PCR (DDPCR), and the presence of the mutation was confirmed by DNA sequencing. Modified cells were characterized by measuring *JAK2* gene expression and the extent of cell proliferation. Interferon α2a (IFN-α2a) and arsenic trioxide were also administered to the cells to explore their potential effects. The *JAK2* V617F-mutated cells were found to exhibit a higher level of *JAK2* gene expression compared with the wild type. Interestingly, a significant increase in the proliferation rate was observed with the modified cells compared with the wild type cells (*p* < 0.001), as assessed from the *JAK2* gene expression levels. Furthermore, the treatments with IFN-α2a and arsenic trioxide led to the preferential suppression of the cell proliferation rate of the K562 expressing mutant *JAK2* cells compared with the wild type cells, and this suppression occurred in a dose-dependent manner(*p* < 0.01). Moreover, the modified cells were able to differentiate into megakaryocyte-like cells following stimulation with phorbol 12 myristate 13 acetate (PMA). Taken together, the results of the present study have shown that the CRISPR/Cas9-modified *JAK2* V617F model may be used as a disease model in the search of novel therapies for MPNs.

## 1. Introduction

Myeloproliferative neoplasms (MPNs) are clonal disorders of multipotent hematopoietic progenitors. *BCR::ABL1*-positive is the hallmark of chronic myeloid leukemia, whereas *BCR::ABL1*-negative MPNs clinically manifest as polycythemia vera (PV), essential thrombocythemia (ET), or primary myelofibrosis (PMF). The pathogenesis of *BCR::ABL1*-negative MPNs is manifested in the overactivation of the Janus kinase/signal transducer and activator of transcription (JAK/STAT) pathway. The most common causes are mutations of the Janus kinase 2 gene (*JAK2*), most commonly the *JAK2* V617F mutation, and, less commonly, exon 12 mutations. The prevalence of *JAK2* mutations has been reported to be approximately 98% in patients with PV, 50–60% in ET, and 55–65% in PMF [1,2]. In terms of the structure of *JAK2* bearing the *JAK2* mutation, the crystal structure of the pseudokinase (JH2) domain of *JAK2* V617F is highly similar to that of wild type JH2, although it exhibits slight differences in the ATP-binding cleft. The somatic V617F mutation arises from a G to T alteration at nucleotide position 1849 in exon 14 of *JAK2*, resulting in the substitution of valine with phenylalanine at codon 617 in the JH2 domain. The V617F mutation abrogates JH2-mediated inhibition, causing constitutive activation of the JAK2 JH1 kinase [3,4]. This has the effect of rendering the mutated hematopoietic stem cells more hypersensitive to hematopoietic growth factors such as thrombopoietin (TPO) or erythropoietin (EPO), resulting in myeloproliferation, which influences the interrelationships among ET, PV, and PMF [5].

Patients with MPNs show clinical manifestations such as blood cell proliferation, which predisposes them to thrombo-hemorrhagic complications, finally culminating in fibrotic marrow failure or acute myeloid leukemia. Current cytoreductive and antithrombotic therapies are able to prevent the episodic complications of MPNs, although, at present, effective treatments to prevent long-term disease progression from occurring are lacking [6]. Articles have previously been published that attempted to study the function of the *JAK2* gene mutation, specifically *JAK2* V617F, both in cell lines and in experimental mice through introducing abnormal genes into the cells [7,8]. However, the method of introducing modified genes into cells described above may only be temporarily effective and nonspecific. Currently, The Clustered Regularly Interspaced Short Palindromic Repeat (CRISPR)/CRISPR-associated protein (Cas) system is widely used for gene editing, as it allows for the specific and accurate modification of genes. Cas9 is a protein that functions as an endonuclease, which is capable of cutting DNA and causing double-strand breaks (DSBs) at specific locations. Subsequently, the cells undergo DNA repair processes, resulting in the generation of gene mutations, as intended. The advantages of the CRISPR/Cas9 system are its efficiency and its ability to precisely target the regions where DSBs occur [9]. Therefore, the CRISPR/Cas9 technique has a strong applicability for creating disease models to study genetic disorders, and it may be utilized as a tool in therapeutic scenarios due to its capacity for precise genome editing [10].

In the present study, the modified *JAK2* V617F cell line was generated using CRISPR/Cas9 ribonucleoprotein (RNP) complex technology in K562 cell lines to compare the effects of the different experiments/treatments on the modified mutant cells with those on the wild type. Differences in the proliferative capabilities of the wild type and mutant cells were studied. Subsequently, interferon α2a (IFN-α2a) and arsenic trioxide were tested for their potential inhibitory effects on mutated *JAK2* cells. The results obtained demonstrated that the cell line carrying the *JAK2* V617F mutation may be used to screen for drugs that preferentially affect neoplastic cells more than normal cells. This may lead to the development of novel targeted therapies for MPNs.

## 2. Results

### 2.1. Generation and Characterization of K562 Cell Lines Expressing Wild Type and Mutated JAK2 Cells

**Figure 1:**
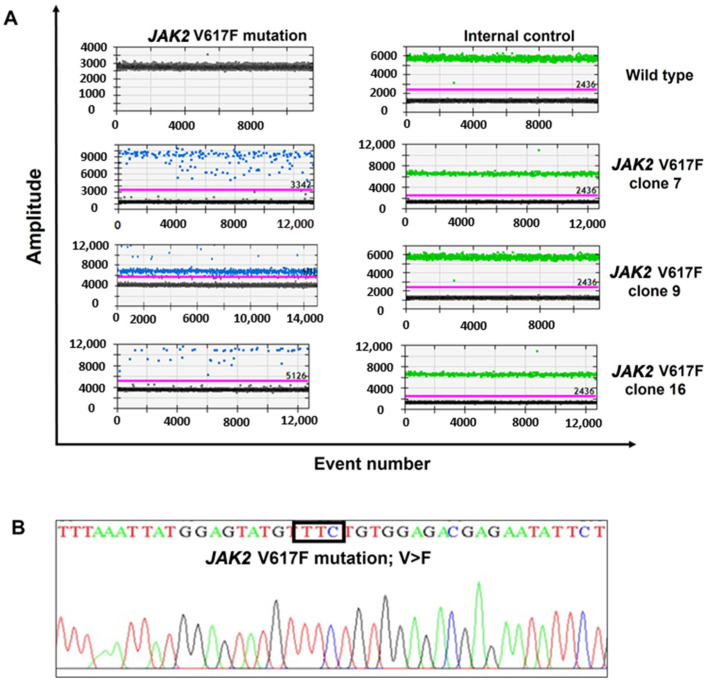
*JAK2* gene mutations in the modified *JAK2* V617F cell lines. (**A**) Droplet digital PCR (DDPCR) expressed *JAK2* V617F mutation (blue dots) and the reference gene (green dots) for testing cells after transfection using CRISPR/Cas9. (**B**) DNA sequencing confirmed the presence of the *JAK2* p.V617F mutation, characterized by a nucleotide substitution from GTC (Valine) to TTC (Phenylalanine) in exon 14, as highlighted in the black box.

The *JAK2* V617F gene mutation was knocked into the wild type K562 cell line using the CRISPR/Cas9 technique. After transfecting the cultured cells for 24 h, the cells were collected and sorted using a flow cytometer. The single cell which exhibited fluorescence was plated in a 96-well plate and subsequently cultured until a sufficient quantity of cells was attained. Twenty fluorescence-positive cells were harvested and subsequently subjected to the extraction of genomic DNA for the analysis of the *JAK2* gene mutation. After edition and selection, droplet digital PCR (DDPCR) was used to detect the *JAK2* gene mutation in cell lines. *JAK2* V617F cell clone numbers 7, 9, and 16 showed both of the signals with the *JAK2* V617F mutation probe (blue dots) and the reference probe (green dots), whereas the wild type cells expressed only the reference probe signals (Figure 1A). DNA sequencing was subsequently used to validate the *JAK2*p.V617F point mutation, representing a substitution of G in GTC (valine) to T in TTC (phenylalanine) in exon 14 of the *JAK2* gene (Figure 1B).

### 2.2. JAK2 Gene Expression and Cell Proliferation

**Figure 2:**
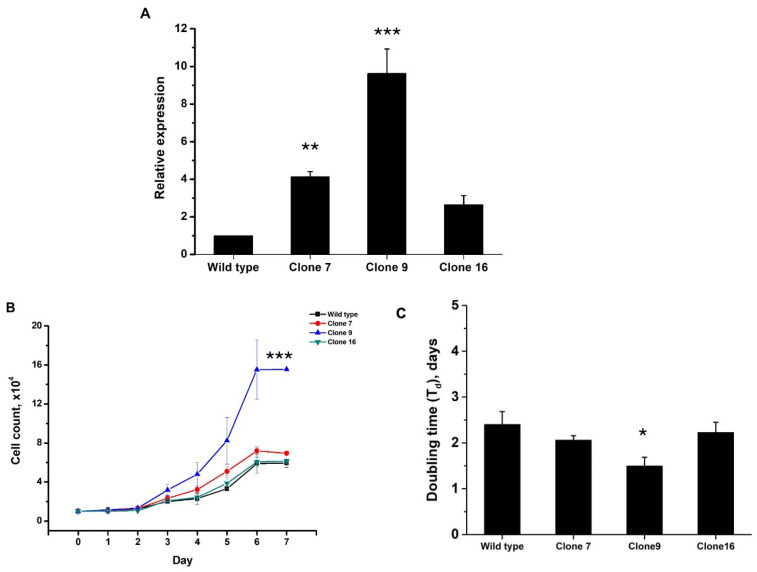
Characteristics of modified cell lines. (**A**) Exogenous *JAK2* gene expression levels in K562 cell line after gene editing compared to wild type. (**B**) Total cell counts from three different cells that exhibited the mutation in the *JAK2* V617F gene. (**C**) The doubling time (T_d_) in days for wild type cells and clones 7, 9, and 16 harboring the *JAK2* V617F mutation. Data are presented as means ± standard deviations (SDs) from three independent experiments. The asterisks (*), (**), and (***) denote *p* < 0.05, *p* < 0.01, and *p* < 0.001, respectively.

The selected cells were evaluated for the gene expression of *JAK2*. *JAK2*-mutated cell clone numbers 7, 9, and 16 were found to express the levels of the *JAK2* gene at approximately 4.13 ± 0.28 fold (*p* = 0.005), 9.63 ± 1.30 fold (*p* < 0.001), and 2.64 ± 0.49 fold (*p* = 0.116), respectively, when compared with wild type (Figure 2A). In order to investigate the cell proliferation rate, cells exhibiting the *JAK2* V617F mutation in the three different clones, i.e., clone numbers 7, 9, and 16, as well as wild type K562 cells, were cultured in RPMI 1640 cell culture medium. Cell counts were performed continuously over a period of 7 days. The doubling time of *JAK2* V617F cell clone 7 was 2.06 ± 0.10 days; for cell clone 9, it was 1.50 ± 0.19 days; and for cell clone 16, it was 2.23 ± 0.22 days. The wild type K562 cells exhibited a proliferation rate of 2.40 ± 0.28 days. Notably, *JAK2* V617F cell clone 9 demonstrated a significantly higher proliferation rate than the wild type cells (*p* < 0.05) (Figure 2C).

### 2.3. Effects of IFN-α2a and Arsenic Trioxide on Cells with the Mutated JAK2 V617F Gene

**Figure 3:**
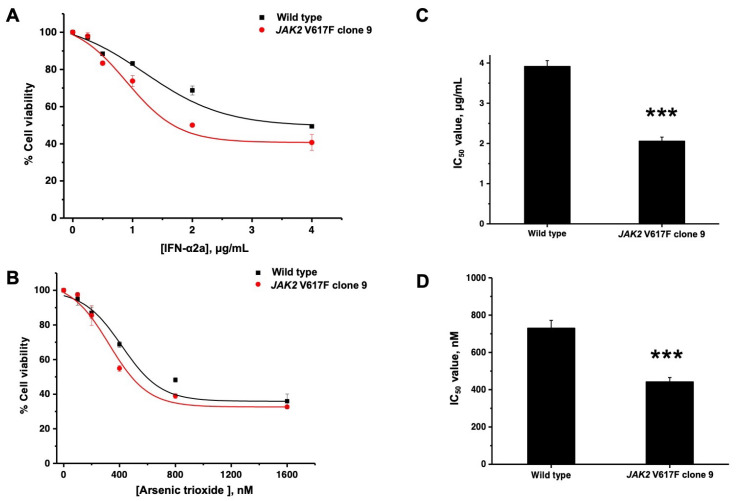
Drug treatment of modified cell lines. Cell viability of *JAK2* V617F clone 9 and wild type after incubations with interferon alpha 2a (IFN-α2a) (**A**) and arsenic trioxide (**B**). The concentration of IFN-α2a (**C**) and arsenic trioxide (**D**) that could inhibit the viability in 50% (IC_50_) compared with wild type. Data are presented as means ± standard deviations (SDs) from three independent experiments. The asterisks (***) denote *p* < 0.001.

IFN-α2a and arsenic trioxide were subsequently administered to *JAK2* V617F cells, specifically clone 9, which demonstrated significantly higher proliferation characteristics compared with the wild type K562 cells, and the treated cells were cultured in RPMI 1640 cell culture medium. The initial cell count was set at 2 × 10^4^ cells/mL in a 96-well plate. Subsequently, either IFN-α2a at a concentration of 0.25, 0.5, 1.0, 2.0, and 4.0 μg/mL, or arsenic trioxide at concentrations of 100, 200, 400, 800, and 1600 nM, was added. The cells were cultured continuously for 48 h, and after completing the incubation period, resazurin solution was added for analysis. The survival percentages of wild type cells following treatment with IFN-α2a at concentrations of 0.25, 0.5, 1.0, 2.0, and 4.0 μg/mL were 97.23 ± 1.84, 88.46 ± 1.07, 83.22 ± 3.02, 72.38 ± 0.27, and 49.36 ± 4.36%, respectively. Meanwhile, those of *JAK2* V617F clone 9 with the same respective concentrations of IFN-α2a were 97.98 ± 1.0, 83.37 ± 0.79, 73.77 ± 0.82, 49.99 ± 2.40, and 40.71 ± 0.70%, respectively (Figure 3A). With respect to arsenic trioxide, the chemical was used to treat wild type cells at concentrations of 100, 200, 400, 800, and 1600 nM, and the resultant survival percentages were found to be 95.04 ± 3.59, 87.03 ± 3.33, 68.77 ± 1.63, 48.20 ± 1.30, and 35.91 ± 4.21%, respectively, whereas the survival percentages of *JAK2* V617F clone 9 for the same concentrations of arsenic trioxide were 97.58 ± 1.05, 85.45 ± 5.67, 54.90 ± 1.83, 38.92 ± 1.42, and 32.63 ± 1.01, respectively (Figure 3B). When calculating the half-maximal inhibitory concentration (IC_50_) values of IFN-α2a and arsenic trioxide, it was found that IFN-α2a at a concentration of 3.92 ± 0.14 μg/mL could inhibit the growth of wild type cells by 50%, whereas for *JAK2* V617F clone 9, the IC_50_ value was determined to be 2.06 ± 0.09 μg/mL. In addition, arsenic trioxide at concentrations of 731.04 ± 40.89 and 443.24 ± 21.65 nM was shown to inhibit 50% of the growth of K562 cells and *JAK2* V617F clone 9 cells, respectively. Importantly, the IC_50_ value of *JAK2* V617F clone 9 when exposed to IFN-α2a and arsenic trioxide was significantly lower than the IC_50_ value of wild type cells (*p* < 0.001) (Figure 3C,D).

### 2.4. Effect of Phorbol 12 Myristate 13 AcetateInduced Differentiation of Modified Cells to Megakaryocytes

**Figure 4:**
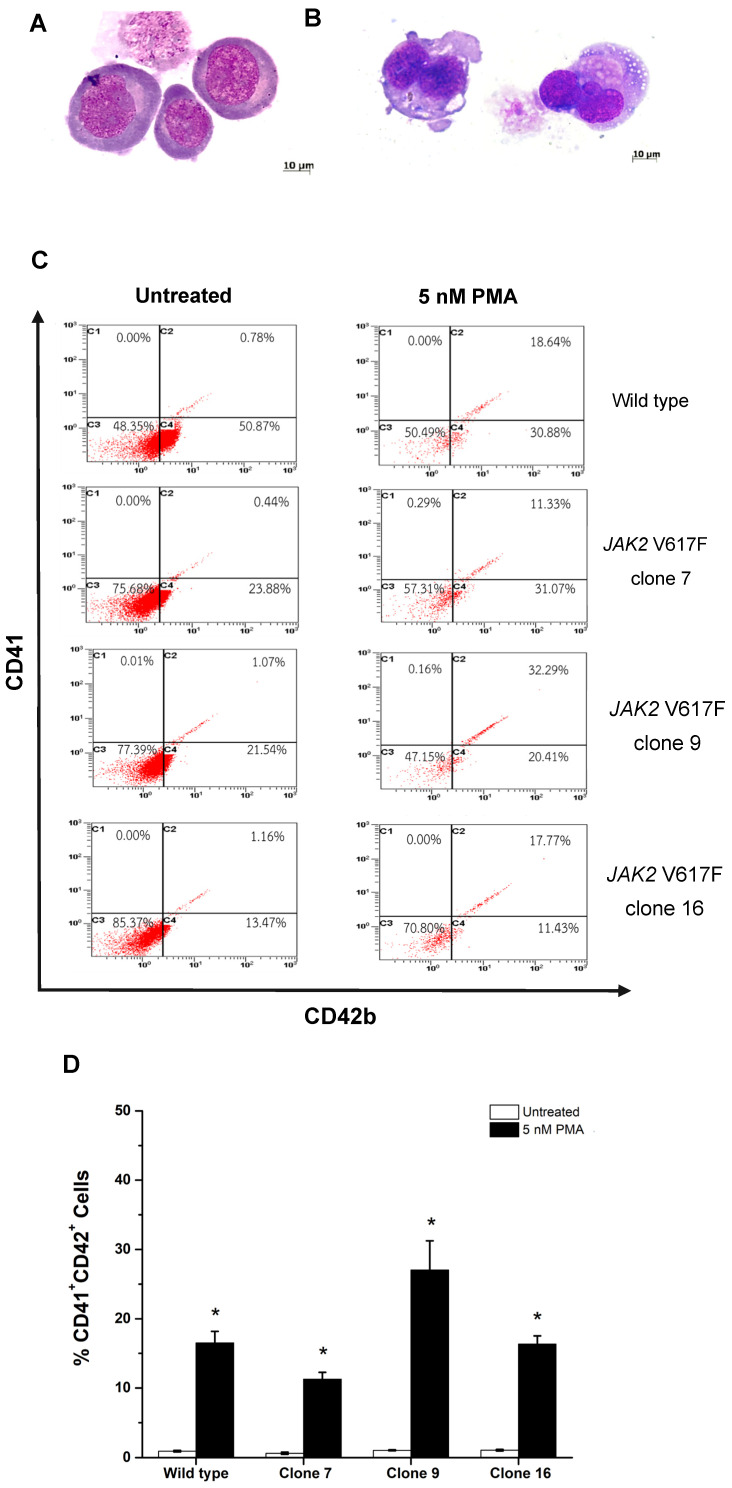
The morphology of cells in a condition without phorbol 12 myristate 13 acetate (PMA) stimulation (**A**) and after induction with 5 nM PMA revealed changes in the cell shape resembling megakaryocytes (**B**). The expression of surface molecules that are specific to the megakaryocyte lineage of cells, *JAK2* V617F clone 7, 9, 16, and wild type, was evaluated both without and with stimulation by PMA (**C**). Percentage of CD41⁺CD42⁺ cells in wild type, clone 7, clone 9, and clone 16 following treatment with 5 nM PMA or under untreated conditions (**D**). Data are presented as means ± standard deviations (SDs) from three independent experiments. The asterisk (*) denotes *p* < 0.05.

After having stimulated cells with 5 nM Phorbol 12 myristate 13 acetate (PMA) for 72 h, wild type cells and cells carrying the mutated *JAK2* gene in three clones exhibited characteristics and a morphology that were similar to the megakaryocyte lineage. It was observed that the cells increased in size and exhibited nuclear lobes division when compared with cells that were not stimulated with PMA (Figure 4A,B). In addition, PMA-stimulated cells expressed CD41/CD42b double-positively on the cell surface at levels of 16.50 ± 1.67, 11.27 ± 0.98, 27.02 ± 4.21, and 16.34 ± 1.18% in wild type, *JAK2* V617F clone 7, 9, and 16 cells, respectively (Figure 4D). The differentiated cells displayed a significant increase in the level of the megakaryocyte marker after induction with PMA when compared with cells without stimulation (*p* < 0.05). Therefore, PMA was shown to be effective at stimulating cells to undergo the changes and transformation necessary to convert them into the megakaryocyte lineage, and these modified cells may be used as a cell model for future studies.

## 3. Discussion

In the present study, the successful construction of a cell line with the *JAK2* V617F mutation using the CRISPR/Cas9 technique has been achieved. The modified cells (especially *JAK2* V617F clone 9) displayed strongly positive fluorescence, with DDPCR detection and confirmation of the point mutation p.V617F in exon 14 of the *JAK2* gene, according to Sanger DNA sequencing. In addition, *JAK2* V617F cells were also used to evaluate *JAK2* gene expression, which was associated with enhanced cell proliferation. In our model, the expression of the mutant *JAK2* gene resembled that of a heterozygous mutation. As a result, the *JAK2* V617F cells specifically exhibited an enhanced differentiation into megakaryocytes following the induction with PMA, similar to what is observed in patients with ET, a condition typically associated with the heterozygous *JAK2*p.V617F mutation.

After confirming the correct mutation of the *JAK2* gene in three clones, 7, 9, and 16, the cell proliferation rates were studied. Interestingly, a significant increase in cell proliferation in the cells from clone 9 was observed with the *JAK2* V617F mutation compared with the wild type; however, no notable differences in cell division were observed between cell clones 7 and 16 and the wild type. This observation suggests that the abundance of the *JAK2* V617F mutation may exert a crucial role in promoting cell proliferation. Based on the DDPCR technique, it was found that *JAK2* V617F clone 9 expressed a higher quantity of the gene, which yielded positive results regarding the specific primer and probe targeting the mutated region. This may be associated with the *JAK2*V617F allele burden, which refers to the proportion of cells with the *JAK2* gene mutation as opposed to those without the mutation. The changing proportions of these cells may be due to the presence of the homozygous or heterozygous forms of the *JAK2* V617F mutation [11]. It has been observed that homozygous gene mutations are often found in patients with PV and PMF, accounting for approximately 30% of cases; however, they are found in only 24% of patients with ET. When cells have a low allele burden, they tend to exhibit phenotypes similar to ET [12], where patients experience high levels of platelets (thrombocytosis). On the other hand, cells with a moderate allele burden tend to exhibit phenotypes similar to PV, where patients experience high levels of red blood cells (erythrocytosis). Finally, cells with a high allele burden tend to exhibit phenotypes similar to PMF, where patients experience high levels of white blood cells (leukocytosis) [13,14]. Furthermore, it has been shown that when cells have a significantly high *JAK2* V617F allele burden, patients may develop PMF, which may then progress to leukemia, along with the presence of constitutive symptoms [11,15].

IFN-α2a has been utilized for treating patients with ET or PV. The effects of IFN-α2a are diverse and include inhibiting cell growth, promoting cell death, blocking blood vessel formation, and modulating the immune system. Interestingly, IFN-α2a can also reduce the levels of mutated *JAK2* alleles in patients with MPNs [16]. Regarding the molecular mechanism of IFN-α2a, it has been reported that IFN-α2a selectively induces apoptosis in *JAK2*-positive cells through the p53 or p38MAPK pathways. Additionally, recent findings have revealed that the sensitivity to IFN-α2a is dependent on STAT2 activation [17]. Arsenic trioxide is the established therapy for relapsed acute promyelocytic leukemia, which exerts its effects through stimulating apoptosis. This involves the interaction of intracellular glutathione and hydrogen peroxide. The suggested ways in which arsenic trioxide acts on other types of cancer involve an increase in the level of reactive oxygen species (ROS) from the mitochondria and/or endoplasmic reticulum, which leads to cellular apoptosis, or programmed cell death [18]. According to the present study, the *JAK2* V617F mutant cells demonstrated a significant decrease in cell viability following treatment with IFN-α2a and arsenic trioxide. Interestingly, IFN-α2a at the concentration of 2.06 μg/mL and arsenic trioxide at the concentration of 443.24 nM specifically inhibited the growth of *JAK2* V617F cells by 50%. Both agents showed a higher sensitivity towards the wild type cells. However, it is advisable to perform cell death assays using Annexin V/propidium iodide or caspase cascade detection to confirm that the cells are indeed undergoing apoptosis [19]. Based on the results of these experiments, the data preliminarily show that IFN-α2a and arsenic trioxide may be utilized as an alternative medication for treating patients with *JAK2* gene mutations, specifically the V617F variant. In the future, the mutant *JAK2*-expressing cells may also be utilized for the evaluation of novel therapeutic agents.

Upon studying the cell morphology and expression of surface molecules specific to megakaryocytes, it has been shown that PMA at a concentration of 5 nM was able to induce the differentiation of K562 cells into megakaryocyte-like cells. This was evidenced by the increased expression of CD41⁺/CD42b⁺ and the presence of larger cell morphology, multiple clumped nuclei and coarse chromatin upon staining in the PMA-stimulated group, resembling megakaryocytes, compared to the non-stimulated group. These findings are consistent with those reported by Huang et al. [20], who demonstrated that treatment of K562 cells with 5 nM PMA for 72 h induced differentiation into megakaryocyte-like cells. The promotion of megakaryocyte differentiation in leukemia cells may occur through an increase in the intracellular levels of reactive oxygen species and the activation of signaling pathways such as the MAPK signaling pathway, including ERK1/2 and p38 [21]. However, it is important to note that PMA stimulation may also induce cell death and, therefore, it is advisable to perform cytotoxicity testing of PMA in order to evaluate its potential harmful effects on the cells [22].

The current study utilized genetically modified K562 cells, a well-established erythroleukemic cell line recognized for its multipotent hematopoietic progenitor-like characteristics. These include a pronounced capacity for erythroid and megakaryocytic differentiation upon specific induction [23]. Such properties have previously established K562 cells as valuable tools for dissecting cellular signaling involved in erythroid and megakaryocytic proliferation processes frequently disrupted in MPNs. Moreover, the intrinsic high transfection efficiency, consistent growth profile, and reproducibility of K562 cells facilitate molecular level studies, particularly beneficial for investigating mutant proteins like JAK2 V617F [24].

In our investigation, we introduced the *JAK2* V617F mutation into K562 cells via targeted genetic engineering, distinguishing this model from naturally occurring MPNs cell lines, such as SET-2, HEL, and UKE-1, which harbor endogenous *JAK2* mutations [25,26]. Notably, while SET-2 cells exhibit intrinsic, spontaneous megakaryocytic differentiation, and HEL or UKE-1 cells depend on external inducers or polyploidization processes [27,28,29], our modified K562 model efficiently undergoes megakaryocytic differentiation specifically following PMA treatment. This targeted differentiation characteristic renders our model particularly suitable for dissecting megakaryocyte-specific signaling pathways.

In terms of pharmacological responsiveness, our modified K562 cells display notable sensitivity to IFN-α, paralleling the responses observed in HEL and UKE-1 cells, yet differing distinctly from SET-2 cells, which predominantly respond to JAK inhibitors [26,30,31]. Thus, our genetically modified K562 cells provide a unique comparative context for studying differential drug responses and therapeutic vulnerabilities within megakaryocytic differentiation pathways pertinent to MPNs. Nevertheless, despite these advantages, it is crucial to acknowledge that K562 cells, originating from an erythroleukemic lineage, do not completely reflect the complex pathophysiological landscape of *BCR::ABL1*-negative MPNs. This intrinsic limitation emphasizes caution in directly translating findings from K562-derived models to the clinical scenarios of MPNs. Therefore, subsequent studies should aim to validate and extend the insights gained from this modified cell line using more clinically relevant cellular models and primary patient-derived specimens. Such approaches will significantly enhance the translational relevance and clinical applicability of the outcomes presented in this research.

## 4. Materials and Methods

### 4.1. Establishment of the K562 Cell Line with CRISPR/Cas9 Expression of the JAK2V617F Mutation

The experimental design using the K562 cell line was approved by the Institutional Review Board of Naresuan University, Phitsanulok, Thailand (certificate no. P10153/63) and was conducted in accordance with the Declaration of Helsinki and International Conference on Harmonization in Good Clinical Practice (ICH GCP). First, the K562 cell line was transfected with the CRISPR/Cas9 ribonucleoprotein complex using an Amaxa™ P3 Primary Cell 4D Nucleofactor™ X Kit (Lonza Group, Köln, Germany). The guide RNA (gRNA) and an oligonucleotide template for genome editing were designed using Alt R™ HDR Design Tool and Templates produced by Integrated DNA Technologies (IDT, Coralville, IA, USA). The gRNA was designed featuring the target 20 base pairs upstream of an NGG PAM sequence, combined with Alt R^®^ CRISPR Cas9 tracrRNA, ATTO™ 550, and Alt R^®^ S.p. HiFi Cas9 Nuclease V3 to form the gRNA/Cas9 protein ribonucleoprotein complexes (the sequences of the gRNA and oligo template are shown in Table 1). After 24 h, transfected cells were sorted using a BD FACSAria II Flow Cytometric Cell Sorter (Becton Dickinson, Franklin Lakes, NJ, USA), and all the K562 cells that expressed fluorescence were sorted as a single cell in a 96-well plate and subsequently cultured in RPMI1640 containing 10% FBS. Cells were incubated at 37 °C with 5% CO_2_ before genomic DNA was extracted to detect the gene mutation using the QIAamp^®^ DNA Mini Kit (Qiagen GmbH, Hilden, Germany).

### 4.2. Characterization of Genetically Modified K562 Cell Lines

The characterization of the genetically modified K562 cells was performed using a DDPCRT100 Thermal cycle. The results were determined using a QX200™ Droplet Reader and QuantaSoft™ Software v.1.6 (Bio Rad Laboratories, Hercules, CA, USA). The positive *JAK2* V617F-mutated cells identified using DDPCR were confirmed to have the *JAK2* gene mutations using Sanger DNA sequencing and analysis via capillary electrophoresis at Macrogen Inc., Seoul, South Korea. In addition, cells were tested for *JAK2* gene expression by using reverse transcription–quantitative PCR (RT-qPCR), following the procedure described in our previous study [32]. The PCR reactions were conducted using the thermocycling protocol: a 95 °C initial denaturation for 10 min, followed by 40 cycles of denaturation (95 °C for 15 s), annealing (59.5 °C for 30 s), and extension (72 °C for 45 s). In order to evaluate the *JAK2* mutation phenotype, the cell proliferation rate of the modified cells was observed for 7 days.

### 4.3. RT-qPCR Analysis

Total RNA was obtained from modified cells by utilizing an RNA purification kit (GeneJET Genomic DNA Purification Kit; Thermo Fisher Scientific, Waltham, MA, USA), and the isolated RNA was reverse transcribed using a cDNA synthesis kit (Thermo Fisher Scientific, Waltham, MA, USA). RT-qPCR assays were performed on an Applied Biosystems™ 7500 Fast real-time PCR system (Thermo Fisher Scientific, Foster City, CA, USA), using Capital qPCR probe mix (Biotechrabbit GmbH, Hennigsdorf, Germany). To standardize the expression levels, the relative quantity of each target gene was compared with that of glyceraldehyde 3 phosphate dehydrogenase (GAPDH), which served as the housekeeping gene. Fold changes were calculated by assessing gene expression using the 2^−ΔΔCq^ method in comparison with normal, unmodified cells. All the samples underwent processing in three independent experiments.

### 4.4. Effects of Drugs on Cell Lines in the Presence of the JAK2 V617F Mutation

IFN-α2a and arsenic trioxide, chemical substances that are used in the treatment of hematological malignancies, were selected for testing, and the effects of both substances were determined with regard to the sensitivity of mutant *JAK2* cells and wild type cells using the phenoxazine dye resazurin. Cells (2 × 10^4^ cells/mL) were cultured in the absence or presence of 0.25, 0.5, 1.0, 2.0, and 4.0 μg/mL of IFN-α2a (Roche, Branchburg, NJ, USA). Arsenic trioxide (M&B, London, United Kingdom) was tested at the concentrations of 100, 200, 400, 800, and 1600 nM. Cells were cultured with the chemicals continuously for 48 h. After the incubation period, resazurin solution at a concentration of 0.1 mg/mL was added and subsequently incubated at a temperature of 37 °C in an atmosphere containing 5% CO_2_ for 3 h. Subsequently, the reaction was assessed using a microplate reader with excitation and emission wavelengths set at 570 and 590 nm, respectively. The percentage of cell viability was determined, and the concentrations of IFN-α2a and arsenic trioxide capable of inhibiting cell viability by 50% (i.e., the IC_50_ values) were calculated using OriginPro 2015 software. All the samples underwent processing in three independent experiments.

### 4.5. PMA-Induced Megakaryocyte Differentiation

To test whether the generated cells could undergo differentiation to megakaryocytes for future use as a cell model, we followed the protocol described by Huang et al. [20] in 2014. *JAK2* V617F and wild type cells were cultured in RPMI 1640 cell culture medium at an initial cell density of 1 × 10^5^ cells/mL and subsequently were treated with PMA at a concentration of 5 nM to induce their differentiation into megakaryocytes. Cells were continuously cultured for 72 h before being prepared for staining. The morphological features of the cells were then observed using Wright–Giemsa staining under a light microscope (Carl Zeiss Microscopy GmbH, Jena, Germany) at x1000 magnification. In addition, the expression level of surface molecules specific to megakaryocytes was studied. Cells were rinsed with PBS and centrifuged at 300 x g for 5 min. After washing, cells were stained with mouse anti-human CD42b PE (BioLegend, San Diego, CA, USA) and mouse anti-human CD41 FITC antibodies (BioLegend, San Diego, CA, USA) that were specific to megakaryocytes. The reactions were allowed to proceed at room temperature in the dark for 30 min, followed by another rinse with PBS and centrifugation at 300 x g for 5 min. The samples were then analyzed for the staining results using a flow cytometer (Beckman Coulter, IN, USA).

### 4.6. Statistical Analyses

A statistical analysis was performed using SPSS software (version 22.0), and the IC_50_ results were extracted using the OriginPro 2015 software. All the data are expressed as the mean ± standard deviation. The Kruskal–Wallis test was also used to detect statistical significances, and *p* < 0.05 was considered to indicate a statistically significant difference.

## 5. Conclusions

Our results have suggested that these mutated *JAK2* V617F cells exhibit a significantly higher rate of proliferation, resembling the characteristics observed in patients with MPNs. Furthermore, when testing the effectiveness of IFN-α2a and arsenic trioxide, it was found that the *JAK2* V617F cells were found to be sensitive to both substances. Therefore, our *JAK2*-mutant expressing cells may serve as a useful model for investigating the molecular mechanisms of *JAK2* mutations and facilitating drug discovery aimed at developing novel therapeutics for MPNs.

## Figures and Tables

**Table 1:** Oligo template and gRNA design for V617F modification using CRISPR Cas9.

Gene	Oligonucleotide Sequences
Guide RNA target 1	AATTATGGAGTATGTGTCTG
Guide RNA target 2	ACGAGAGTAAGTAAAACTAC
Oligo template	TGATGAGCAAGCTTTCTCACAAGCATTTGGTTTTAAACTATGGGGTATGTTTCTGTGGAGACGAGAGTAAGTAAAACTACAGGCTTTCTAATGCCTTTCT

## Data Availability

The data sets generated and/or analyzed during the current study are available from the corresponding author upon reasonable request.

## Abbreviations

The following abbreviations are used in this manuscript:

JAK2	Janus kinase 2
MPNs	Myeloproliferative neoplasms
IFN-α2a	Interferon α2a
PV	Polycythemia vera
ET	Essential thrombocythemia
PMF	Primary myelofibrosis
TPO	Thrombopoietin
EPO	Erythropoietin
Cas9	CRISPR-associated protein 9
DSBs	Double-strand breaks
RNP	Ribonucleoprotein
IC_50_	The half-maximal inhibitory concentration
ROS	Reactive oxygen species
DDPCR	Droplet digital PCR
GAPDH	Glyceraldehyde 3 phosphate dehydrogenase
PMA	Phorbol 12 Myristate 13 Acetate
SD	Standard deviations
gRNA	guide RNA
IDT	Integrated DNA Technologies
HDR	Homology-directed repair
RT qPCR	Reverse transcription quantitative polymerase chain reaction
